# Outcome of Manuscripts Rejected From Intensive Care Medicine: An *In Silico* Study

**DOI:** 10.1186/2197-425X-3-S1-A23

**Published:** 2015-10-01

**Authors:** G Citerio, C Marzorati, JF Timsit, A Perner, J Bakker, M Bassetti, D Benoit, JR Curtis, GS Doig, M Herridge, S Jaber, L Papazian, MJ Peters, P Singer, M Smith, M Soares, A Torres, A Vieillard-Baron, E Azoulay

**Affiliations:** Università degli Studi di Milano Bicocca, Monza, Italy; Università degli Studi di Milano Bicocca, Milano, Italy; Inserm/Univ Paris Diderot, Sorbonne Paris Cite, Paris, France; University of Copenhagen, Copenhagen, Denmark; Erasmus MC University Medical Center, Rotterdam, Netherlands; Azienda Ospedaliera Universitaria Santa Maria della Misericordia, Udine, Italy; Ghent University Hospital, Ghent, Belgium; University of Washington, Seattle, United States; University of Sydney, Sydney, Australia; University of Toronto, Toronto, Canada; Saint Eloi University Hospital, Montpellier, France; Aix-Marseille Universite, Marseille, France; UCL Institute of Child Health and Great Ormond St Hospital, London, United Kingdom; Tel Aviv University, Tel Aviv, Israel; University College London Hospitals, London, United Kingdom; D'Or Institute for Research and Education, Rio de Janeiro, Brazil; Hospital Clınic of Barcelona, Barcelona, Spain; Hospital Ambroise Pare, Paris, France; Paris Diderot Sorbonne University, Paris, France

## Introduction

In 2013, Intensive Care medicine received 1516 manuscripts, with a global acceptance rate of 20.7%. Among the full length articles (original articles, narrative review, systematic review/meta-analyses, my paper 20y later), 1093 were original articles and 10.07% were accepted.

## Objectives

This study is part of the quality control of the ICM editorial's line. Rejected papers were tracked to know in which journal and after which time these were finally accepted.

## Methods

On February 2015, a Pubmed search was performed to identify journals and publication dates for original articles rejected by ICM in 2013. Articles were considered as “published on another journal” if they met the following criteria: similar title, same first author, similar abstract, and publication year ≥ 2013. Articles were classified in 4 categories:

1. not published;
2. published in the same impact factor journal ( ± 0.5);
3. published in higher impact factor journal or
4. published in lower impact factor journals.

## Results

There was no statistical correlation between submitting country and group categories. Among the 983 original articles analyzed, 485 (49.3%) were published in other Journals, with a mean ( ± SD) time to publication of 6,9 ± 3,02 months after ICM's rejection. All but 17 articles that were published elsewhere were rejected without review. 19 (4%) articles were published in journals with same IF, 14 (3%) in journals with higher IF and 440 (91%) in journals with lower IF. Twelve articles initially rejected by ICMwere finally published in ICM as letters to the editor.

Table 1 reports time from submission to ICM's decision between different manuscript's categories, as well as time from ICM's rejection to final publication.

**Table 1:** Table 1

	Articles	Average delay submission - first decision (days ± SD)	Median delay submission - first decision (days)	Average delay ICM's rejection - final publication (weeks ± SD)
Higher IF	14	12.71 ± 11.66 #	8	7.71 ± 3.65
Same IF	19	2.53 ± 3.34	1	6.88 ± 3.5
ICM	12	6.08 ± 8.64	2	2.5 ± 1.73
Lower IF	440	7.61 ± 10.89	2	7.02 ± 2.99

Thirteen of the rejected manuscripts (2.6%) received more than 5 citations from January 2014 to January 2015. of these, 8 were classified in the lower IF category, 2 in the same IF, 2 in ICM, and only 1 in the higher IF category. Conversely, 80 articles among 110 published by ICM during 2013 (72.7%) received more than 5 citations in the same period. Sensitivity and specificity for an article to receive more than 5 citations if accepted by ICM editorial staff was respectively 85.11% and 81.71%.

## Conclusions

About a half of ICM rejected articles were published on other journals, mostly with lower IFs. Only a very small proportion of rejected articles received a number of citations higher than ICM's IF, and it relates with the interval needed for the first decision.

